# Supplementary material for: A Facile Approach to Solid-State White Emissive Carbon Dots and Their Application in UV-Excitable and Single-Component-Based White LEDs
Source: Nanomaterials (Basel). 2019 May 10;9(5):725. doi: 10.3390/nano9050725 (PMC6566165; doi:10.3390/nano9050725)
Supplement: Supplementary file 1 [file nanomaterials-09-00725-s001.pdf]

Supporting Materials

A Facile Approach to Solid-State White Emissive Carbon Dots and Their Application in UV-Excitable and Single-Component-Based White LEDs

Xiangyu Feng [1,2], Kai Jiang [2], Haibo Zeng [1,*] and Hengwei Lin [2,*]

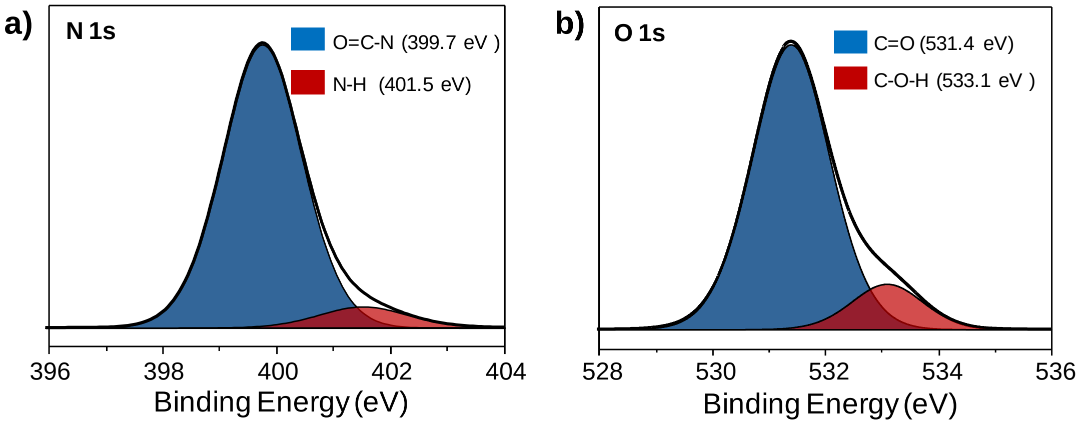

Figure S1. The high resolution XPS N1s (**a**) and O1s (**b**) spectra of the W-CDs powder and their corresponding deconvoluted fitting results.

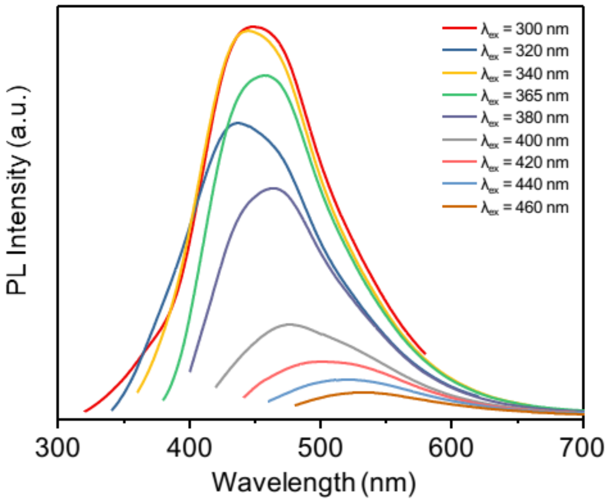

Figure S2. PL spectra of the W-CDs aqueous dispersion under various excitation wavelengths. The excitation wavelengths ranged from 300 nm to 460 nm with an increment of 20 nm. The concentration of W-CDs was 0.5 mg mL^{-1}.

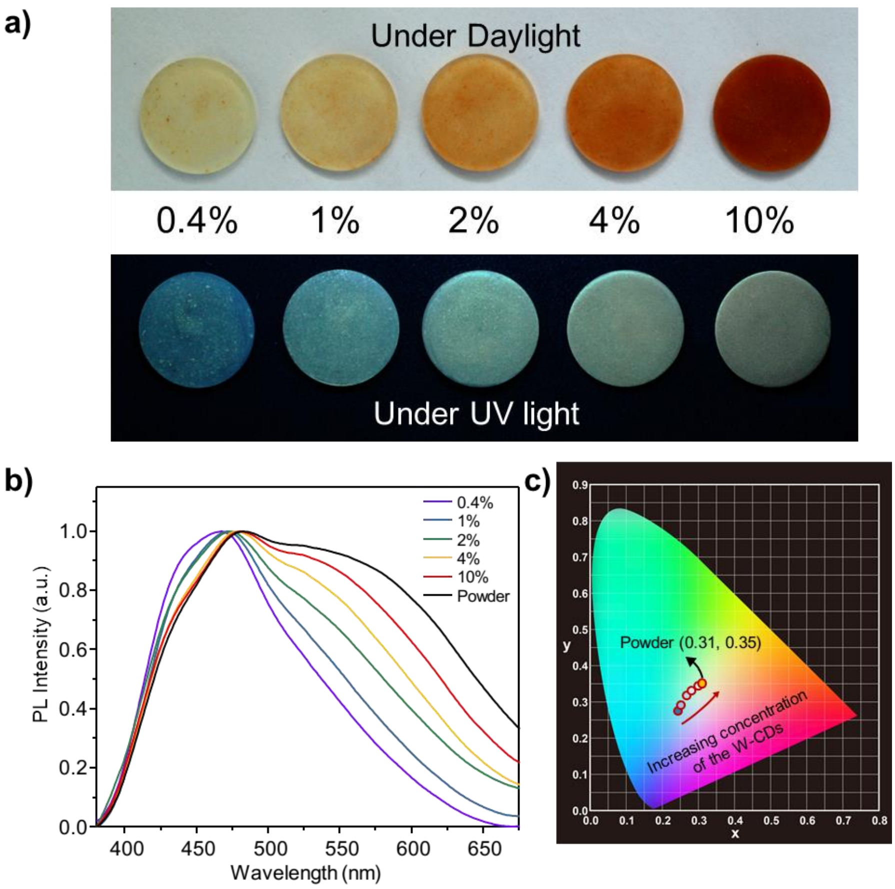

Figure S3. (**a**) Photographs of the W-CDs@epoxy resin composite films under daylight and UV excitation (365 nm). (b-c) Normalized PL (**b**) and CIE 1931 chromaticity coordinates (**c**) of W-CDs@epoxy resin films with various W-CDs concentration (mass ratio against epoxy resin) under the UV excitation (365 nm).

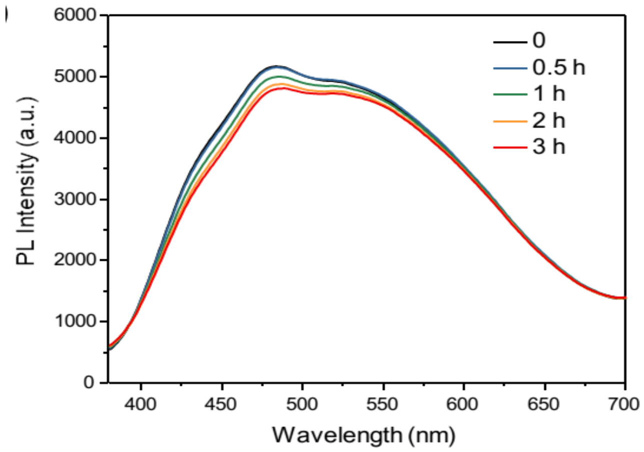

Figure S4. PL spectra of the W-CDs@epoxy resin lighting film with 10% material contained (mass ratio) under continuous UV (365 nm, 250 W Xe lamp) irradiation.

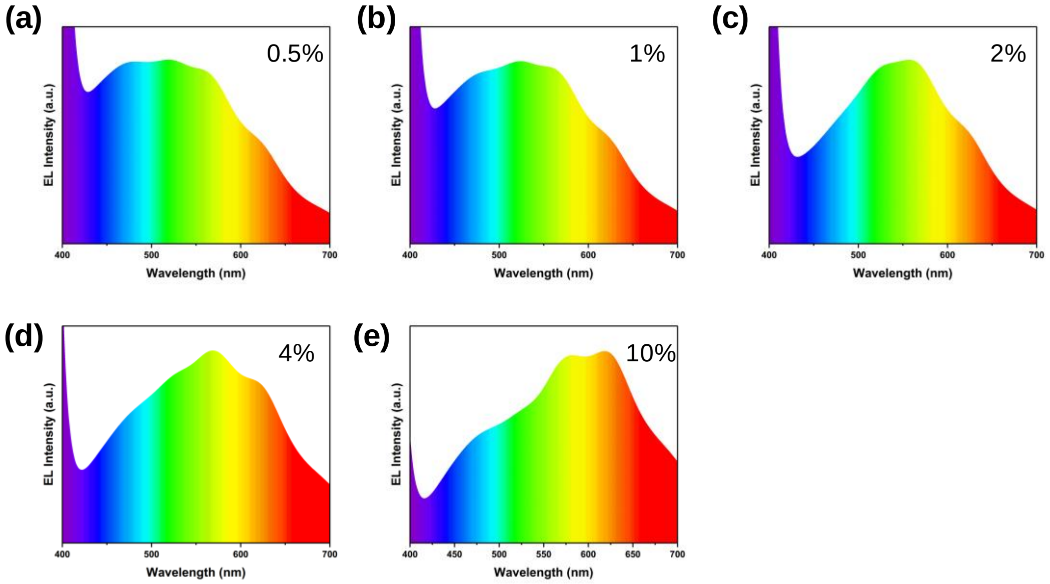

Figure S5. (a-e) EL spectra of the W-LEDs with various W-CDs mass concentration in color conversion layer.

Table S1. Relative contents of various chemical bonds in the W-CDs based on XPS measurements and fitting results.

Type of bonds	C 1s				O 1s		N 1s	
	C-C/C=C	C-O	C-N	N-C=O	C=O	C-O-H	amide N	amino N
Relative Contents (%)	30.79	9.83	16.07	43.31	87.14	12.86	92.35	7.65

Table S2. Fitting parameters of the PL lifetimes of the W-CDs powder and aqueous dispersion.

Sample	λex/ nm	λem/ nm	τ1/ ns	B1/ %	τ2/ ns	B1/ %	Avg/ ns	CHISQ
W-CDs powder	319	482	1.75	65.51	7.07	34.49	2.36	1.729
W-CDs dispersion (in water)	319	456	2.89	31.07	11.2	68.93	5.92	1.346

Table S3. Reported CDs with self-quenching-resistance and performances of their corresponding LEDs.

Emission Color.	Reference	SSLCDs			WLEDs				
		**QY (%)**	**λ_{ex} (nm)**	**Color**	**λ_{ex} (nm)**	**L(cd/m^2)**	**(X, Y)**	**CRI**	**CCT**
Single-color	Ref[s1]	/	340	Yellow	365	/	(0.29, 0.34)	/	/
	Ref[s2]	8.5	365	Red	365	/	(0.31, 0.31)	/	/
	Ref[s3]	1.04<br>0.42	/<br>/	Yellow<br>Orange-red	365	4993	(0.29, 0.30)	83	8526
White	Ref[s4]	2.0	365	White	365	/	(0.31, 0.30)	76.2	7034
	**This work**	**6.7**	**365**	**White**	**365**	**6002**	**(0.30, 0.35)**	**83**	**6897**

References

S1. Chen, Y.; Zheng, M.; Xiao, Y.; Dong, H.; Zhang, H.; Zhuang, J.; Hu, H.; Lei, B.; Liu, Y. A Self-Quenching-Resistant Carbon-Dot Powder with Tunable Solid-State Fluorescence and Construction of Dual-Fluorescence Morphologies for White Light-Emission. *Adv Mater* **2016**, *28*, 312-318; DOI: 10.1002/adma.201503380.

S2. Shao, J.; Zhu, S.; Liu, H.; Song, Y.; Tao, S.; Yang, B. Full-Color Emission Polymer Carbon Dots with Quench-Resistant Solid-State Fluorescence. *Adv Sci (Weinh)* **2017**, *4*, 1700395; DOI:

10.1002/advs.201700395.

S3. Feng, T.; Zeng, Q.; Lu, S.; Yan, X.; Liu, J.; Tao, S.; Yang, M.; Yang, B. Color-Tunable Carbon Dots Possessing Solid-State Emission for Full-Color Light-Emitting Diodes Applications. *Acs Photonics* **2017**, *5*; DOI: 10.1021/acsphotonics.7b01010.

S4. Jiang, B.P.; Yu, Y.X.; Guo, X.L.; Ding, Z.Y.; Zhou, B.; Liang, H.; Shen, X.C. White-Emitting Carbon Dots with Long Alkyl-Chain Structure: Effective Inhibition of Aggregation Caused Quenching Effect for Label-Free Imaging of Latent Fingerprint. *Carbon* **2018**, *128*, 12-20; DOI: 10.1016/j.carbon.2017.11.070.
